# Feasibility of Early Meal Detection Based on Abdominal Sound

**DOI:** 10.1109/JTEHM.2019.2940218

**Published:** 2019-09-11

**Authors:** Konstanze Kölle, Anders Lyngvi Fougner, Reinold Ellingsen, Sven Magnus Carlsen, Øyvind Stavdahl

**Affiliations:** 1 Department of Engineering CyberneticsNorwegian University of Science and Technology (NTNU)7491TrondheimNorway; 2 Department of EndocrinologySt. Olavs University Hospital7491TrondheimNorway; 3 Department of Electronic SystemsNorwegian University of Science and Technology (NTNU)7491TrondheimNorway; 4 Department of Clinical and Molecular MedicineNorwegian University of Science and Technology (NTNU)7491TrondheimNorway

**Keywords:** Abdominal sound, artificial pancreas, bowel sounds, meal detection, pattern recognition

## Abstract

In classical approaches for an artificial pancreas, continuous glucose monitoring (CGM) is the only measured variable used for insulin dosing and additional control functions. The CGM values are subject to time delays and slow dynamics between blood and the sensing location. These time lags compromise the controller’s performance in maintaining (near to) normal glucose levels. Meal information could enhance the control outcome. However, meal announcement by the user is not reliable, and it takes 30 min to 40 min from meal onset until a meal is detected by methods based on CGM. In this pilot study, the use of bowel sounds for meal detection was investigated. In particular, we focused on whether bowel sounds change qualitatively during or shortly after meal ingestion. After fasting for at least 4 h, 11 healthy volunteers ingested a lunch meal at their usual time. Abdominal sound was recorded by a condenser microphone that was attached to the right upper quadrant of the abdomen by medical tape. Features that describe the power distribution over the frequency spectrum were extracted and used for classification by support vector machines. These classifiers were trained in a leave-one-out cross-validation scheme. Meals could be detected on average 10 min (std: 4.4 min) after they had started. Half of these were detected without false alarms. This shows that abdominal sound monitoring could provide an early meal detection. Further studies should investigate this possibility on a larger population in more general settings.

## Graphical abstract

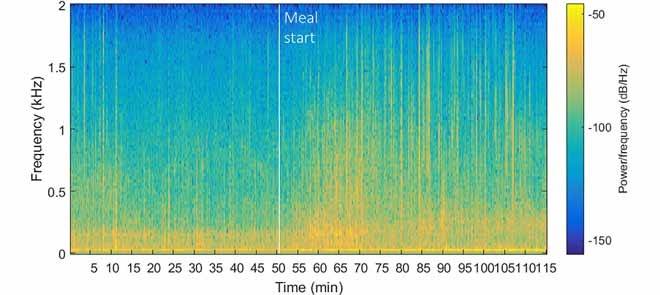

## I. Introduction

### A. Meal Detection in an Artificial Pancreas

The pancreas produces insufficient insulin in diabetes mellitus type 1 (DM1). Persons suffering from DM1 are dependent on exogenous insulin to regulate the blood glucose level (BGL). The manual insulin therapy is a time-consuming daily task that often dominates the life of affected people. To reduce this burden, worldwide research efforts focus on the development of a so-called artificial pancreas, a fully automated system that controls the BGL [1]. The most advanced systems rely on continuous glucose monitoring (CGM) by sensors that are placed in the subcutaneous (SC) tissue. Based on the CGM value (along with static parameters such as body weight, insulin-to-carbohydrate ratio, insulin sensitivity, active insulin time and glucose target range), algorithms decide on the amount of insulin that will be injected into the SC tissue. The slow glucose kinetics and slow insulin absorption in the SC tissue limit the glucoregulatory outcomes of these SC systems by limiting the bandwidth of the closed loop and thereby causing e.g. larger postprandial glucose excursions. [2].

To reduce the postprandial BGL while avoiding episodes of serious hypoglycemia, meal announcements are required by clinically tested systems for glucose control [2]. Such requirements do not disengage the user from the mental occupation with the disease. A more aggressively tuned controller may be able to mitigate increased BGL after meals by administering insulin more liberally. This in turn increases the risk of postprandial hypoglycemia, which single-hormone systems have no way of mitigating effectively, potentially resulting in a dangerous situation. For a successful mitigation of postprandial hyperglycemia, insulin should be administered as soon as possible. Automated detection of meals has therefore been studied, which could trigger an increased insulin administration. Approaches for automated meal detection proposed so far exploit the CGM data of SC sensors, e.g. [3], [4]. The delay between meal start and reliable meal detection can easily reach 40 min [5]–[7] although our recent studies show that it may be possible to reduce this detection time significantly [8], [9]. The fusion of measurements from two redundant SC sensors was proposed to enhance the reliability [10], but alternative sensing modalities have not been exploited.

This paper investigates the use of audible signals from the gastrointestinal tract for automated meal detection. To the knowledge of the authors, Al Mamun and McFarlane (2016) [11] were the first to suggest the integration of recordings of abdominal sounds into an artificial pancreas. They developed a portable bowel-sound detector system.

The aim of this work is to study whether abdominal sounds are changing significantly enough during and shortly after meal intake to allow a reasonably early detection for use in an artificial pancreas.

## II. Background

### A. Characteristics of Bowel Sounds

Bowel sounds are non-stationary, transient events. They can be differentiated into two main types: (a) clicks of short duration occurring alone or in sequences, (b) clusters of (non-differentiable) bursts of longer duration, e.g. [12]. The typical frequency range of bowel sounds lies between 50 Hz and 1500 Hz [13]–[15]. Single studies report maximum frequencies of up to 3000 Hz [16] or 5000 Hz [17]. However, the power spectrum density above 1500 Hz is rather low (see Fig. 5 in [16]). A more recent study confirmed that only 0.5 % of the signal’s power spectrum density occurs at frequencies above 1000 Hz [18]. The same study revealed that the largest part of the power spectrum density of abdominal sounds is located between 100 Hz and 500 Hz [18], while a minimum frequency of 80 Hz has been chosen by others before [14].

**FIGURE 1.**
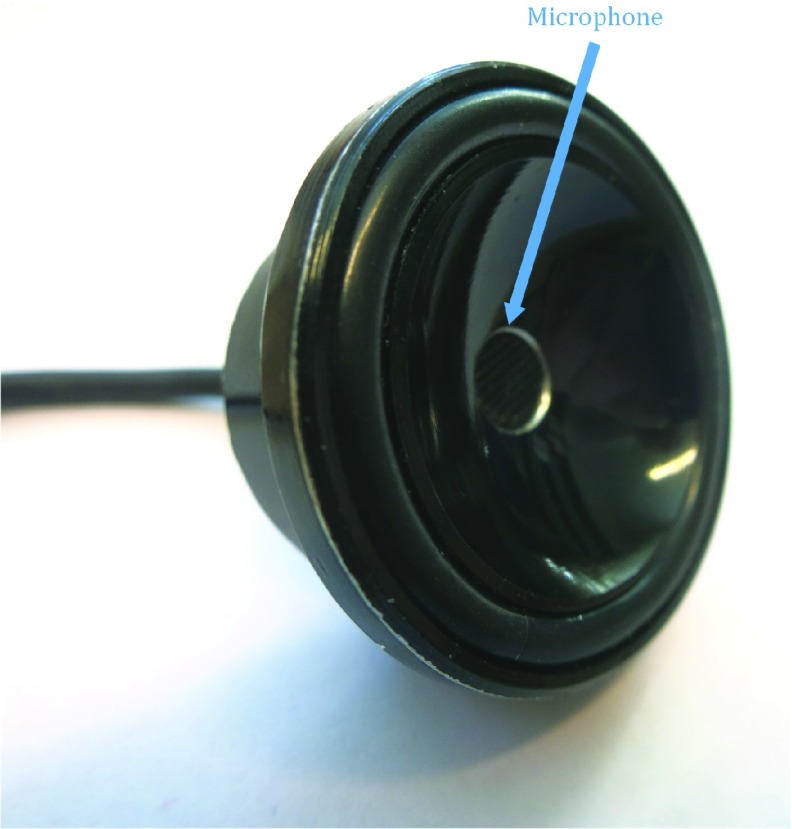
Chestpiece from a stethoscope with microphone in the center hole.

**FIGURE 2.**
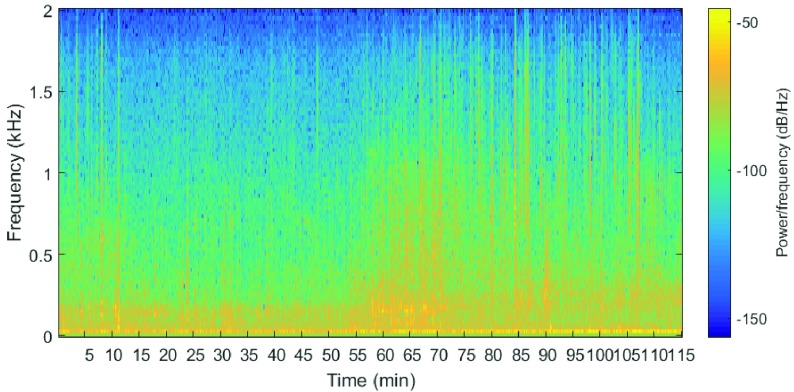
Spectrogram of a recording in the training set. Windows with a length of 20 s and overlap of 10 s were analyzed using spectrogram function in MATLAB. The meal started at minute 51.

**FIGURE 3.**
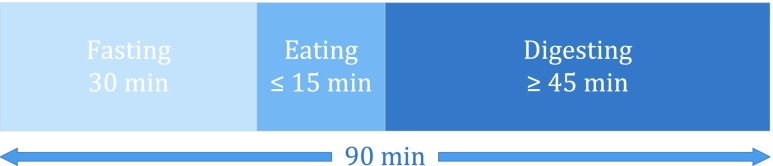
Protocol of recording sessions in pilot study.

**FIGURE 4.**
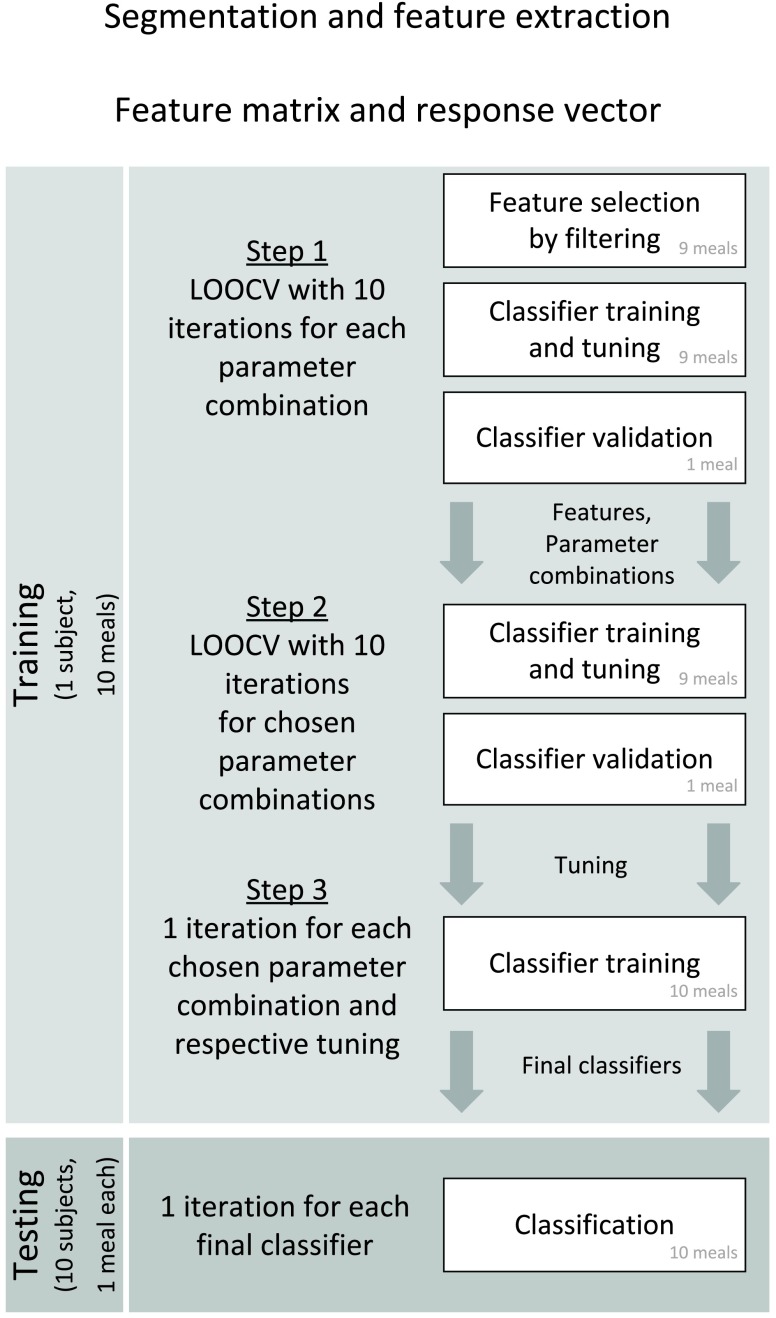
Procedure for training (Section IV-C) resulting in the final classifier that is tested on the test set (Section IV-D). The term “LOOCV” indicates leave-one-out cross-validation. The generated output of each step that is used in the following is defined between the downward pointing arrows. Classifier validation is always evaluated based on the meal detection performance.

**FIGURE 5.**
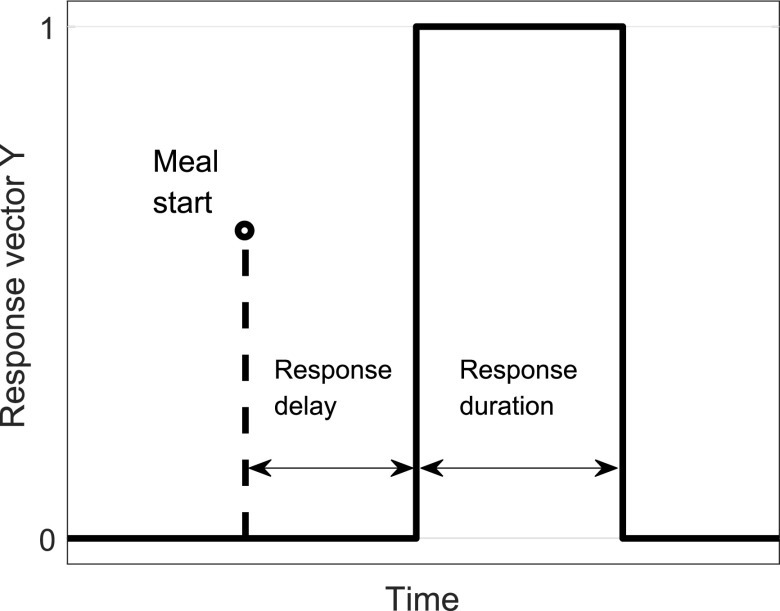
Definition of the parameters *response delay* and *response duration* relative to the meal start. Instances with values of “1” and “0” in vector $Y$ (Eq. 3).

### B. Measures of Bowel Activity

Early studies report the number of bowel sounds per time unit, the duration of bowel sounds and duration of silence to describe the bowel activity [16], [19], [20]. Hansen and Haslinger (1984) [21] analyzed the signal power in different frequency bands. More recent studies combine features based on both occurrence rate of bowel sounds and their frequency distribution in the analysis of abdominal sound [18], [22].

### C. Pre- vs. Postprandial Sounds

Abdominal sound has been studied with focus on the extraction of stationary sound patterns to diagnose gastric disorders. The dynamics of bowel sounds have received less attention. Previous studies that compared pre- and postprandial sounds did not cover the actual period of the meal intake but rather paused the recording (or at least did not report results for the prandial period) [17]–[19], [23]–[26]. Other studies mention the analysis of pre- and postprandial recordings, but do not report the timing relative to the meal onset [16], [24].

## III. Experimental Set-Up

### A. Recording

A Sennheiser MKE2 P-C condenser microphone (Sennheiser Electronic GmbH & Co. KG, Wedemark, Germany) was fixed in the chest-piece of a classical stethoscope, as shown in Fig. 1. Single-channel 24-bit audio signals with a sampling frequency of 32 000 Hz were recorded using a the digital audio recorder 722 (Sound Devices LLC, Reedsburg, Wisconsin, US). The data was analyzed on a personal computer using MATLAB and Signal Processing Toolbox Release 2016b (The MathWorks, Inc., Natick, Massachusetts, United States).

### B. Pre-Processing

Before further processing, the raw signals were down-sampled to a frequency of 4000 Hz using the Chebyshev Type I IIR filter of order 8 as anti-aliasing filter. This accelerates processing without loss of information since the maximum frequency of bowel sounds is not higher than 2000 Hz.

The amplification of the signals varies between the different recordings. For meal detection, we are interested in detecting a characteristic change within the signals. To be able to combine recordings with different amplification for training and tuning, each recording was linearly mapped to the range [−1, 1]. By this normalization, the relative change within the signals is considered, rather than their absolute amplitudes. All reports of signal power in the following thus relates to the normalized signals.

### C. Data Sets

#### 1) Training Set

The idea of using bowel sounds for early meal detection was initially investigated on one subject. This volunteer was neither diagnosed with nor had self-reported gastrointestinal disorders. In 17 of the in total 18 available recordings, the subject sat reclined on a chair, whereas the subject was lying during the remaining recording. Recordings with less than 15 min of fasting state prior to the lunch were excluded. In total, 10 lunch meals of this subject were included in the training set. Each lunch consisted of wheat-ray bread with cheese. Breakfast (müsli, fruits, dairy) had been finished 4.5 h to 5 h before the lunch started. The recorder’s amplification was tuned from one recording to the other in order to find a suitable amplification that would be high enough to grasp sounds of low power in rather silent periods, and low enough to not saturate when loud bowel sounds would occur later on. Adjusting the amplification at the beginning of the recording was rather challenging, because recordings usually started without significant bowel sounds, while louder sounds occurred later. The amplification was not adjusted during recordings as the sound power envelope was expected to contain useful information. The microphone was fixed in the upper right quadrant of the abdomen in all but two recordings. In those two recordings, the lower right quadrant was used. After fixation of the microphone by medical tape, the microphone was covered by clothes. This was done to increase the comfort of the subject during the more than one-hour recordings.

Figure 2 provides the spectrogram of one recording in the training set that shows the frequency distribution over time. It was created by plotting the short-time Fourier transform of windows that had a length of 20 s and were overlapping by 10 s. This example illustrates that the initial recordings on one subject suggested that the gastrointestinal sound activity increased in the early digesting phase compared to the fasting.

The aim of the present study is to investigate whether the described differences can be utilized to detect meals early enough to be beneficial in an artificial pancreas.

#### 2) Test Set

Based on the initial one-subject study, the experimental procedure for the pilot study was refined: the amplification was chosen rather low to avoid saturation; a seated but reclined posture rather than lying flat was adopted for maximum comfort of the participants.

Ten volunteers were enrolled in the pilot study, which was approved by the Regional Ethical Committee Central, REK Midt 2018/28. All subjects were self-reported healthy with respect to gastrointestinal functions.

Subjects were asked to keep their regular life style at the days prior to the recording. This included the timing, amount and composition of meals, as well as type and intensity of physical activities. On the day of recording, they had breakfast as usual and then fasted until lunch. The recording sessions were scheduled at the typical lunch time of each subject to match the individual alimentary habits. Furthermore, subjects brought their own usual lunch meal. The microphone was fixed in the upper right quadrant of the abdomen using medical tape. This quadrant was chosen to maximally capture sounds originating from the region where the stomach ends in the duodenum.

Figure 3 depicts the protocol of a session: The recording started in fasting state. After 30 min, the subjects started to eat and used maximum 15 min to finish their meal. The meal was followed by an at least 45 min long digesting period. The recording ended after 90 min in total.

During the recording, the subjects remained reclined with the instruction to move as little as possible. The food was positioned in an easily reachable distance from the subject. “Unforeseen events” such as heavy movements while changing the posture, coughing, nose-clearing, or exogenous disturbances from the surroundings were logged by the subjects, who were also asked not to use electronic devices that could interfere with the recording device. Periods where subjects reported “unforeseen events” such as heavy movements were discarded.

## IV. Methods

The training and testing procedure is illustrated in Fig. 4. The proposed meal detection is based on the binary classification of a feature matrix. This feature matrix must be constructed before the classification can take place. The transformation from the recorded audio signals into feature matrix and response vector is described in Sections IV-A and IV-B. Sections IV-C and IV-D deal with the training and testing, respectively. When evaluating the performance, one must differentiate between classification and meal detection. Section IV-E defines the performance measures that are applied during training and testing.

### A. Segmentation and Feature Extraction

The original signal was segmented before features were extracted for each segment. Segments of length 10, 20, 30, and 60 s were implemented as Table 1 shows. Successive segments were overlapping by half of their length.

**TABLE 1:** Parameter Variations in Feature Matrix $X$ and Response Vector $Y$. $Response ~Delay$ and $Response~ Duration$ are Illustrated in Fig. 5

Parameter	Values	Unit
Length of overlapping segments in $X$ and $Y$	10, 20, 30, 60	s
Response delay in $Y$ (relative to meal start)	0, 2, 4, 6, 8, 10	min
Response duration in $Y$	1, 2, 5, 10, 15, 20	min

Features were chosen based on the spectral analysis in Section III-C. They are listed in Table 2. The spectral analysis indicated that the power distribution over the frequency range changes shortly after a meal intake starts. In particular, the power below 1000 Hz is increasing. Frequencies above 1000 Hz were included because one could visually differentiate between noise and increased sonic bowel activity based on the power distribution: Noise occurred often over the whole frequency range, while bowel sounds caused a power increase mainly up to 1000 Hz. If a silent period is followed by bowel sounds, one would thus expect an increase of power below 1000 Hz. Typical noise, on the other hand, would cause an increased power level including the frequency range between 1000 and 2000 Hz. Bands of 100 Hz width between 0 and 2000 Hz were therefore considered. For each segment $s$, an estimate of the power spectral density $\hat {P}_{s}(f)$ dependent on the frequency $f$ in this segment $x_{s}$ was generated:

\begin{equation*} \hat {P}_{s}(f) = \frac {\Delta t}{N} \Bigl |\sum _{n=0}^{N-1}x_{s,n} e^{-i2\pi f n} \Bigr |^{2}\tag{1}\end{equation*}

with $\Delta t$ the sampling interval, $N$ the number of samples in segment $s$, for $-1/2 \Delta t < f \leq 1/2 \Delta t$. This estimate was then integrated to compute both the total power and the power in specific frequency intervals. For each time segment, the power fraction within each frequency interval was generated by division of the power in the particular frequency interval by the total power.

**TABLE 2:** Extracted Features in Each Segment

Feature No.	Description
1	Total power in the frequency range from 0 Hz to 2000 Hz
2 to 21	Power in 100 Hz-wide bands from 0 Hz to 2000 Hz
22 to 41	Power fraction in 100 Hz-wide bands from 0 Hz to 2000 Hz

### B. Feature Matrix and Response Vector

All features were collected in the feature matrix $X$:

\begin{equation*} X = \begin{bmatrix} f_{1}(1) & f_{2}(1) & \cdots & f_{N}(1)\\ f_{1}(2) & f_{2}(2) & \cdots & f_{N}(2)\\ \vdots & \vdots & \ddots & \vdots \\ f_{1}(K) & f_{2}(K) & \cdots & f_{N}(K) \end{bmatrix} \tag{2}\end{equation*}

with feature $f_{n}(k)$, $n = 1,\ldots,N$, in segment $k = 1,\ldots,K$. One row represents all $N$ features in one segment, while the vertical dimension represents time. Its extent depends on the length of the recordings. Each feature column $n$ was processed by a median filter with 2 min width to mitigate occasional outliers. This smoothing was introduced because in particular the shorter segments were subject to single outlying peaks, and we were interested in the average increasing power. The median-filtered features were linearly scaled to the range [0, 1] [27] for each feature individually.

Closely related to the feature matrix is the response vector. Each row in $X$ (Eq. 2) was assigned to either the class “1” or the class “0.” This assignment is defined in the response vector $Y$:

\begin{equation*} Y = \left [{0, \ldots, 0, 1, \ldots, 1, 0, \ldots, 0}\right ]^{T}, \tag{3}\end{equation*}

with the class “1” as meal indicator and “0” as default. The class “1” does not necessarily coincide with the period in which the subject actually ate nor does its onset always coincide with the meal start. It rather defines the instances that are set to 1 in the response vector. Figure 5 provides an illustration of the class assignment in the response vector $Y$ relative to the meal start. The *response delay* was introduced to consider the possibly delayed onset of audible bowel activity after the meal start. The *response duration* was varied to find the interval length best suited for early meal detection. The implemented values of *response delay* and *response duration* are summarized in Table 1.

Feature matrices and response vectors were constructed with all combinations of the three parameters in Table 1. The term “parameter combination” in the following refers to any of these 144 parameter combinations.

When several recordings were used for training, their features were linearly scaled for each recording separately to consider the individual differences in general power. The scaled features were vertically stacked in $X$. This extends the time dimension while the number of features stays the same. The response vector $Y$ was extended in the same way.

### C. Training

The goal of the training procedure is a final classifier that is thereafter tested on an unknown data set. Figure 4 illustrates that the training is composed of three steps. During these three steps, the training set is used which consists of 10 meals from the same subject (Section III-C.1). The output of each step builds the basis of the next step and is indicated between the downward pointing arrows.

Step 1: In the first step, features were selected before a support vector machine (SVM) classifier^1^ was trained and validated. This was repeated for each of the 144 parameter combinations in Table 1. The motivation for this supervised learning strategy was that different features may be relevant depending on the definition of “1” in the response vector $Y$ (see Fig. 5). Consequently, we applied leave-one-out cross-validation (LOOCV) throughout the whole training procedure [28] in step 1. For each parameter combination in Table 1, a LOOCV with 10 iterations was performed where each of the 10 meals was used for validation once. To ensure that the trained classifier was not biased towards the validation meal, it was left out during feature selection and classifier training. Each of the LOOCV iterations in step 1 included the following:
  1a. The features of 9 training meals were composed in the feature matrix $X$.
  1b. The feature matrix was input to a feature selection by filtering: The correlation between each feature and the meal response vector was calculated and checked against a threshold of 10 %, which is computationally efficient and yields a conservative result (c.f. Section VI). Features whose correlation did not exceed this threshold were discarded.
  1c. The remaining features in $X$ together with the response vector $Y$ were then used to train SVM classifiers with radial basis function. A grid with 2^[−10,−5,0,5,10]^ for both the box constraint and the kernel scale was used to perform tuning by grid search. One SVM model was built for each grid point. The classifier tuning that resulted in the lowest mean squared error between the classifier output and the response vector of the validation meal was chosen.
  1d. Whereas the sample classification was used to tune the classifier in 3., the meal detection of the validation meal was compared to eventually choose the classifiers.
  The results of training step 1 illustrated the general feasibility (see Section V). Those features and parameter combinations that resulted in the highest number of TP detected validation meals were chosen and forwarded to step 2. The feature sets chosen for the same parameter combination in the different LOOCV iterations differed slightly. In order to conclude with a single classifier for each parameter combination, the feature sets chosen for each parameter combination in step 1 were combined in step 2.
Step 2: One SVM model was built for each of the most promising parameter combinations. The following LOOCV procedure was applied in training step 2 to find the final tuning for each chosen parameter combination:
  2a. The feature matrix $X$ was composed of the features of 9 training meals.
  2b. Feature selection was omitted. Instead, all features were included that had been selected for this parameter combination during one of the LOOCV iterations in training step 1.
  2c. An SVM model with radial basis function was built based on the selected features and the response vector. The same grid search as before was applied to tune the SVM classifier.
  2d. The classifiers were applied to classify the validation meal resulting in a classification error for each LOOCV iteration.
  The classification errors of the 10 LOOCV iterations were averaged for each chosen parameter combination and tuning. The tuning with the lowest average error was selected for the specific parameter combination.
Step 3: In training step 3, the classifiers with the tuning chosen in step 2 were trained once more on the whole training set of 10 meals to include as much variability as possible.

^1^Support vector machines are classifiers that can be applied for overlapping classes with non-linear decision boundaries. A more detailed description of support vector machines is beyond the scope and thus omitted here. See for example [28] for an in-depth introduction.

### D. Testing

The final classifiers built in Section IV-C were tested on 10 meals, each of a different subject (Section III-C.2). This test set had not been used in any of the training steps. The results of meal detection for the test set is reported in Section V-D.

### E. Performance Measures

a: Performance of Classification

The classification can be evaluated based on the sample prediction error. The sample prediction error is the fraction of misclassified observations in the training, validation or test set, respectively.

In meal detection for fully automated glucose control in DM1, the outcome of a false classification is asymmetric: When a meal is not detected it has no consequences other than that the postprandial period will be poorly regulated by the nominal control system. A false meal detection, on the other hand, could trigger a fatal insulin injection and cause hypoglycemia. This calls for an analysis that explicitly accounts not only for true positive detections (i.e. detected actual meals), but also false positives (i.e. detection of a meal where no meal takes place). Based on the elements of the confusion matrix (true positive (TP), false negative (FN), false positive (FP), and true negative (TN), cf. Table 3), the true positive rate (TPR) and the false positive rate (FPR) are determined as follows:

\begin{equation*} TPR = \frac {TP}{TP+FN} \,, \tag{4}\end{equation*}

and:

\begin{equation*} FPR = \frac {FP}{FP+TN} \,. \tag{5}\end{equation*}

TPR and FPR can be used to generate a so-called Receiver-Operator Characteristic (ROC) plot as in Figs. 7 and 8, which is a common way to evaluate classification results. In this graph, the main diagonal represents mere chance (akin to a coin toss), while a perfect binary classifier is represented by the upper left corner (i.e. TPR = 1, FPR = 0). Consequently, the further above and to the left of the diagonal a given system is placed, the better it performs.

**TABLE 3:** Illustration of Results in Binary Classification

		Assigned class	
		1	0
True class	1	TP	FN
	0	FP	TN

**FIGURE 6.**
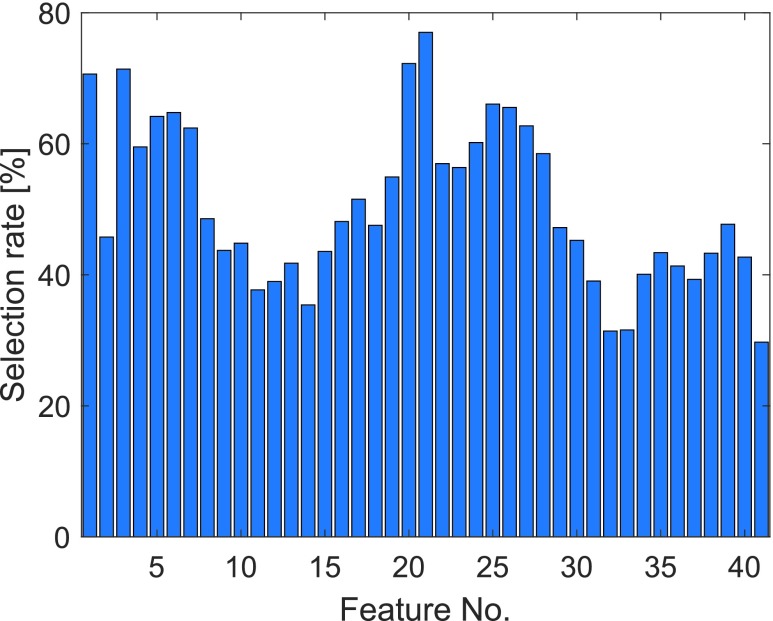
Selection rate of features by filtering in training step 1. The fraction relates to the runs where at least one feature is selected. Features are numbered according to Table 2.

**FIGURE 7.**
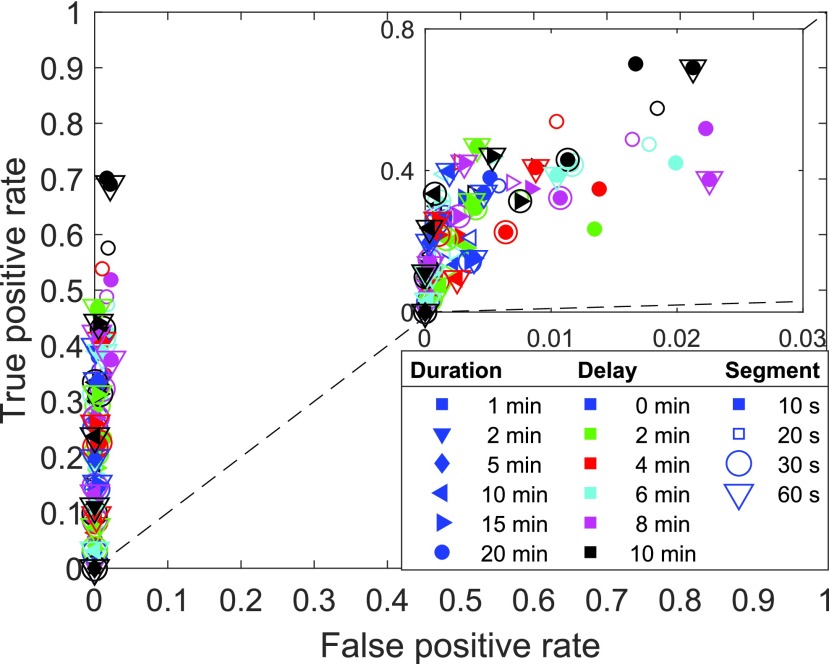
Sample classification of the training meals in training step 1 (Section IV-C). True positive rate vs. false positive rate. One marker represents the average of all 10 LOOCV runs with the same parameter combination. The overall performance is illustrated while the exact distribution of the data points is not important. The parameter combinations with the best classification performance are magnified part in the upper right corner.

**FIGURE 8.**
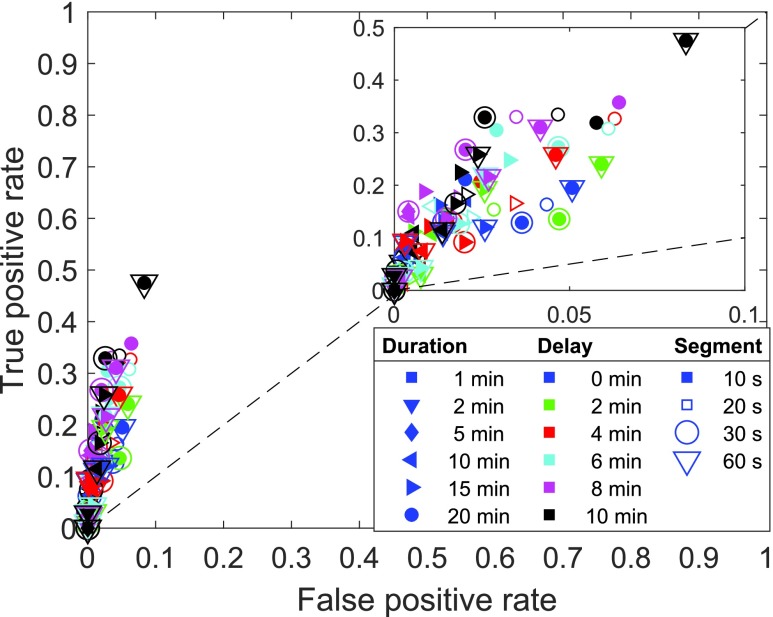
Sample classification of the validation meals in training step 1 (Section IV-C). True positive rate vs. false positive rate. One marker represents the average of all 10 LOOCV runs with the same parameter combination. The overall performance is illustrated while the exact distribution of the data points is not important.

b: Performance of Meal Detection

For meal detection in the context of an artificial pancreas, it is not critically important whether each *sample* is classified correctly. As soon as a meal is detected, this information will be processed by a superior control layer that decides about an insulin dosing. Instead of a performance measure based on samples, we therefore introduce a meal-based measure: A TP meal detection occurs if two successive samples within 30 min after the meal start are assigned to the class “1.” This TP meal duration does not coincide with the eating period but considers that the audible bowel activity may increase delayed to the start of food intake.

A FN meal detection is accordingly counted if no two successive class “1” samples occur within the 30 min period.

All samples classified as “1” outside of the positive meal period could be defined as FP detection. However, once a meal started, the remaining part in the data set belongs to the digestion. Furthermore, a repeated meal detection within the digestive period is no drawback but rather a confirmation of the previous meal detection. Supervisory control decisions (that are out of the scope of this pilot study) could be based on enhanced certainty about the meal. No FP is therefore assigned for detection after the meal start. Prior to the meal, false detections can occur. Again, only if two consecutive samples indicate a false detection, it is counted as such in this study. These definitions of TP and FP are introduced to assess the performance of meal detection in the present data set. A supervisory controller in a real implementation, however, does not know whether a detection is TP or FP and trigger the same control actions.

An evaluation of TN meal detections along these lines does not add value to the analysis and is therefore omitted.

Besides the sensitivity towards meals, the time delay between meal start and detection is important when this information shall be used in an artificial pancreas. The time of meal detection is reported relative to the meal start (marked by the dashed bar with circle in Fig. 5) when the person started eating. The response delay in Fig. 5 is introduced for training purposes to consider the possibly delayed onset of bowel sounds. However, the timing of the response vector in the training set does not influence the reported time of meal detection.

## V. Results

### A. Feature Selection

The feature selection is performed for each of the parameter combinations in Table 1 in training step 1. Figure 6 presents the selection rate per feature. The most frequently selected features are: Power in the ranges 1800 Hz–2000 Hz (features 20 and 21) and roughly 0 Hz–700 Hz (features 3–7 and 22–28), as well as the total power (feature 1). It indicates that these frequency bands of the recorded sound contain the most important information with regards to early meal detection.

### B. Parameter Selection

This paragraph relates to Step 1 of Figure 4. The selected features are used in each LOOCV run to train and validate an SVM model. This is repeated for each parameter combination. Figure 7 is an ROC plot (cf. Section IV-E.a) of the classified samples in the training meals in training step 1. Different marker properties describe the parameter combination: The shape indicates the response duration in the response vector $Y$; the color indicates the response delay in $Y$ relative to the meal start; unfilled and filled markers, and markers surrounded by a circle or triangle indicate the length of the segments. Each parameter combination is represented by one marker that summarizes the results of 10 LOOCV runs by averaging. The achieved maximum TPR is 0.7 for a response duration of 20 min, a response delay of 0 min and a segment length of 10 s (black, filled, not surrounded circle). Most parameter combinations result in TPRs of less than 0.5. At the same time, the FPR is comparatively low with a maximum of 0.023. This demonstrates that abdominal sounds contribute with substantial information about meal intake.

The training set is, however, not suited to evaluate the performance of the classifier. Instead, Fig. 8 shows the TPR vs. FPR averaged over all meals that are left out for validation. As expected, the classification of the validation sets results in fewer TP samples and more FP samples. The TPR is less than 0.5 for any of the parameter combinations while the maximum FPR increases to 0.08, which still confirms substantial information about meal intake. All markers are still located on the left side of the identity function’s graph (the dashed diagonal line in the figure), i.e. more samples are classified TP than FP when validating the trained classifiers.

### C. Meal Detection in Training Set

**FIGURE 9.**
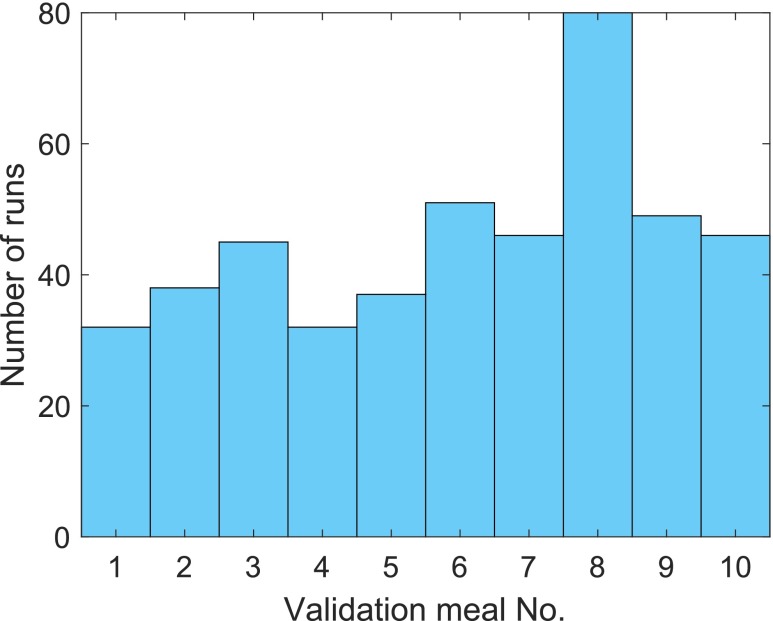
Number of runs per validation meal in training step 1 (Section IV-C) that resulted in meal detection without false alarm (TP = 1, FP = 0).

The classification of samples is used to detect meals according to the criteria in Section IV-E. At best, the meal is detected within 30 min without prior false detection. The number of classifier validations in training step 1 with this ideal outcome (TP = 1, FP = 0) is shown in Fig. 9. The maximum number is 144 because each of the meals in the training set is once left out in the 10 LOOCV runs for each of the 144 parameter combinations. For each left-out validation set, at least 32 parameter combinations and tunings of the trained classifiers existed that led to successful meal detection. 80 runs with validation meal No. 8 had one TP and no FP.

**FIGURE 10.**
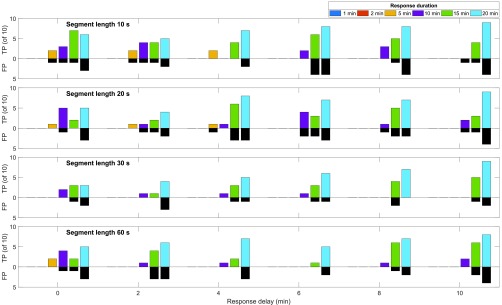
Meal detection of the sets used to validate the classifiers in training step 1 (Section IV-C). Number of true positive and false positive meal detections for different parameter combinations.

Figure 10 compares the number of TPs and FPs per parameter combination in training step 1. The colored bars turned upwards indicate the number of true positives; the black bars turned downwards indicate the number of false negatives. According to the definitions in Section IV-E, the maximum of both is 10. The three shortest response durations (1, 2, and 5 min – dark blue, red, yellow) are not or only rarely presented in Fig. 10. In most runs with a response duration of 1 min or 2 min, no feature is selected such that no classifier could be trained and no meal detected. Despite a successful feature selection, a response duration of 5 min resulted in poor classification performance. Even a response duration of 10 min appears to be too short with the current set-up. A response duration of 20 min shows the best result with up to 9 TP meals and only 2 false alarms. This is achieved with both a segment length of 30 s and a response delay of 10 min. The second best performance with 9 TPs and 4 false alarms is achieved with segments of 10 and 20 s length, and a response delayed by 10 min in $Y$.

**FIGURE 11.**
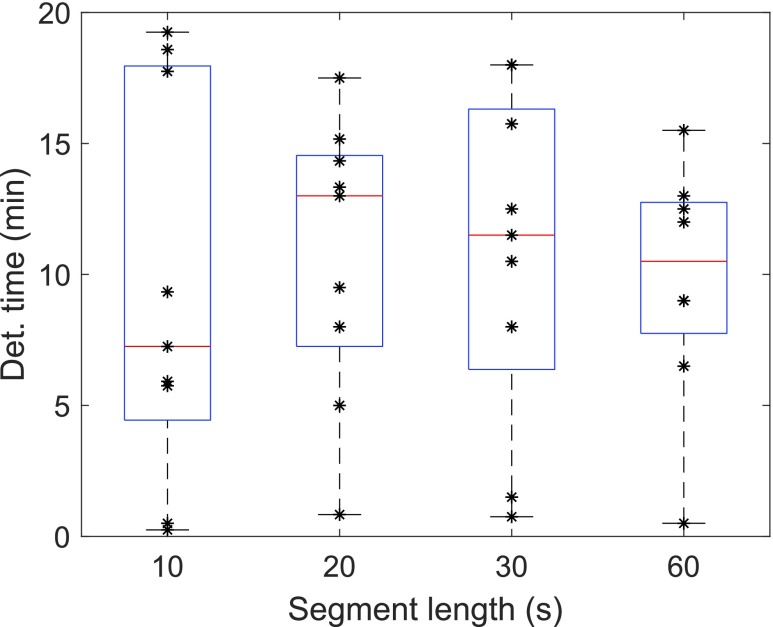
Detection time of the validation meals used to validate the classifiers in training step 1 (Section IV-C). Boxplots of meal detection in minutes after meal start for runs with a response duration of 20 min and a response delay of 10 min. The stars indicate the time of detection for the single validation meals. The red line is the median detection time. Mean values are given in Table 4.

In the context of an AP, it is important how long after the actual start of food intake the detection occurs. The time of detection is defined as the second TP sample. Table 4 presents the time of meal detection in minutes from the meal start for chosen parameter combinations. Runs with the same parameter combinations are averaged in this table, excluding runs where the meal is not detected. With segment lengths of 10 s, it took on average less than 10 min to detect the meals, and around 1 min to 1.5 min more with segment lengths of 20, 30, and 60 s. While shorter segments yield faster meal detection, we expect longer segments to produce classification results with a higher likelihood of being correct. However, even a delay of 11 min is significantly smaller than what is exhibited by comparable meal detection methods. The variation between the validation meals is visually illustrated by the boxplots in Fig. 11. If a validation meal is detected, it is so within 1 min to 20 min, which is well within the performance range of contemporary meal detection methods. The wider distributed detection times with the 10 s-segment cf. Fig. 11 indicate that this segment length might be less suited than for example the 60 s-segments with the narrowest distribution. However, given the limited size of our dataset, this result can hardly be stated with any statistical significance.

**TABLE 4:** Detection Time of the Validation Meals Used to Validate the Classifiers in Training Step 1 (Section IV-C). Mean and Standard Deviation of Meal Detection After Meal Start for Runs With a Response Duration of 20 Min and a Response Delay of 10 Min (cf. Fig. 5)

Segment length (s)	10	20	30	60
Mean (min)	9.6	11.1	11.2	10.8
Standard deviation (min)	7.5	5.4	6.4	4.7

Based on these results of training step 1, the parameter combinations of the final SVM models are chosen: for all segment lengths, a response delay of 10 min and a response duration of 20 min in the response vector. Classifiers with these parameter combinations are tuned in a LOOCV scheme in step 2, before the final classifiers are built in training step 3.

### D. Meal Detection in Test Set

**FIGURE 12.**
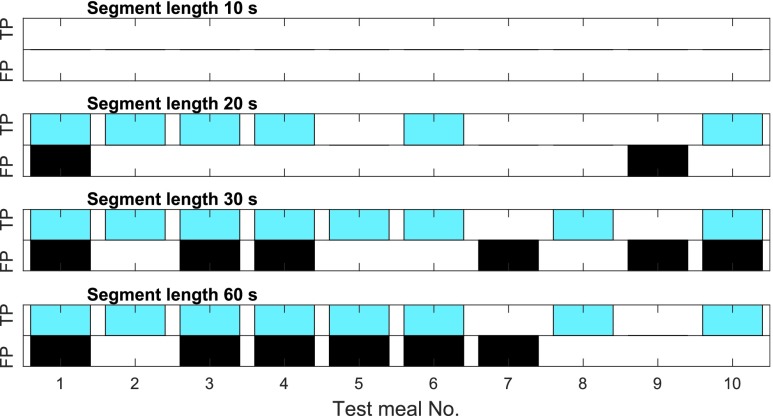
Meal detection of the test set (Section IV-D) with the four final classifiers. True positive and false positive meal detection for each test meal.

The four final classifiers are tested on the previously unused test set which contains one meal from ten different subjects. The results of meal detection are presented in Fig. 12. None of the test meals are detected using a segment length of 10 s. The set-up with 20 s-segments results in 6 detected meals while 2 false alarms occur. For the 30 and 60 s-segments, 8 meals are detected along with 6 false alarms. Considering only meal detections without false alarm (TP = 1, FP = 0), a segment length of 20 s is most successful in this set-up.

**FIGURE 13.**
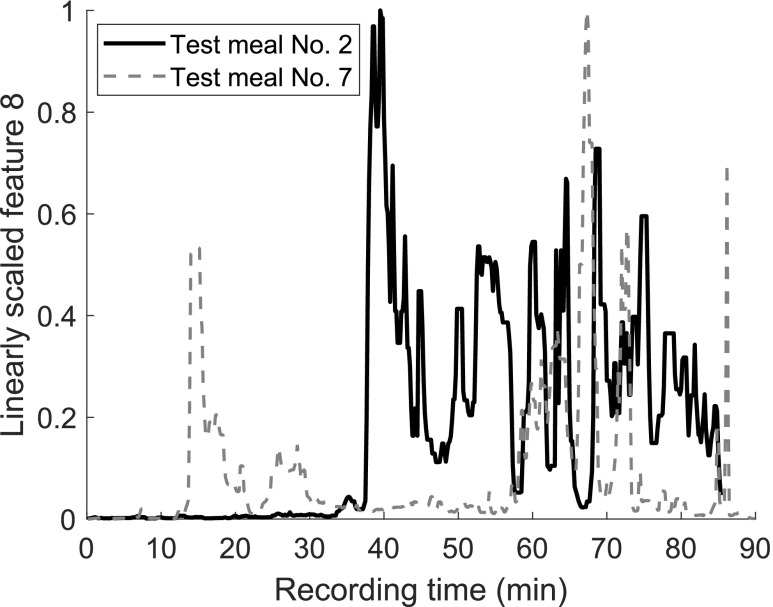
Example of variation between subjects in test set. Feature 8, power in frequency band from 300 to 350 Hz, for a segment length of 20 s. The meal starts at minute 30.

For simplicity, in the following paragraph the meal of each test subject is referred to as a “test meal,” although strictly speaking the subject is the variable. The number of successful detection varies between the test meals.Confusing terminology. Calling it test meal sounds like the meal is the variable. Either this terminology should be formally introduced prior to use, or a different terminology should be used. While test meal 2 is successfully detected with three final classifiers, test meals 7 and 9 are not detected with any set-up. If test meal 1 is detected, it causes each time also a false alarm. The variation within the test set is illustrated in Fig. 13. The curves show the linearly scaled feature 8 (power in frequency band from 300 Hz to 350 Hz) for test meals 2 and 7. The power in this frequency band increases abruptly 8 min after test meal 2 started. Instead of such a significant change shortly after the meal start, the recording of test meal 7 includes two periods of increased power: one before the meal, the other starting about 30 min after the meal. Three other test meals are followed by a clear increase in relevant frequency bands similar to test meal 2. The pattern of test meal 7 occurs less distinct in three other recordings, but with a shorter first period that is not as often erroneously detected as meal. The recording of test meal 1 contains almost no variation. Test meal 9 is contaminated by significant background noise that masks the changes caused by bowel activity.

Neglecting test meals 1, 7 and 9, the classification of the remaining test meals with a 20 s-segment length results in 5 of 7 TP meal detections without false alarm. The average time of detection for these meals is 8.4 min with a standard deviation of 5.6 min.

## VI. Discussion and Future Work

The present pilot study demonstrates that abdominal sound recordings can potentially be used for meal detection in an artificial pancreas. Lunch meals were investigated in 11 healthy subjects. A larger population should be studied to confirm the generality of the findings. Eventually, people with DM1 should be enrolled because they may suffer from gastrointestinal disorders as a consequence of the primary disease. The system needs to work for them, since they are the target users of an artificial pancreas.

low yield, poor result The findings of this study are promising: With the best combination of parameters (response duration, response delay and segment length, cf. Fig. 5), it took on average only 8.4 min (std: 5.6 min) from the onset of the meal to its detection (cf. last paragraph of Section V-D). By comparison, meals have previously been detected based on CGM data from the SC tissue within 30 min to 40 min after meal onset [5]–[7]. Postprandial insulin delivery after 10 min, compared to 30 min to 40 min, would markedly reduce glucose elevations without increasing the risk of late postprandial hypoglycemia. The average diurnal glucose control would also be improved.

As this is a feasibility study, the conclusion will be qualitative, not quantitative. A true positive rate of 0.4–0.5 and a false positive rate of up to 0.08 may be unacceptable in a practical AP system, since a false prediction and a following insulin bolus may cause serious hypoglycemia. Nevertheless, the study demonstrates that abdominal sounds contribute with substantial information about meal intake earlier than indicated in previous studies. This bears promise that sound analysis, possibly combined with other complementary sensor modalities, can constitute an important part of a future system for early meal detection. Such a system may be exploited e.g. for closed-loop control or as a “meal reminder” for patients who commonly forget meal boluses.

The number and the rate of occurrence of bowel sounds have previously been linked to the state of digestion [18]. The spectrograms in Fig. 2 indicate differences in gastrointestinal sound activity between the preprandial and early postprandial period. Based on this observation, the total power and the power fraction in different frequency intervals were extracted as features. However, the power estimates are independent of the presence of bowel sounds in the segments. An increased sound power around meal time may partly even be caused by meal-related activities (such as moving arms and hands, scratching cutlery, etc.). Typical artifacts cause a different power distribution over the frequency range than bowel sounds which can be exploited to eliminate artifacts [18], [29]. Motivated by this, power ratios were included as features in this study. Furthermore, those sounds are related to eating, and as such they could be regarded as valid indicators of food intake. However, the recordings were performed in an environment with relatively few potential noise sources. The task of detecting changes in audible bowel activity will be more challenging in free-living situations. For this reason, the present results should be regarded as an indication of feasibility and by no means as quantitatively conclusive.

The general audible bowel activity differed between the recorded meals in the training set. In some recordings one can clearly hear bowel sounds from the beginning, while in others they are less prominent or even absent. The reasons for this difference can be two-fold: First, the number and duration of bowel sounds may differ markedly between recording sessions in the same subject as well as between different subjects. This is likely because of natural physiological variations. Second, the recording condition differed in terms of amplification due to the recorder-internal amplifying factors, the contact pressure between skin and microphone, the exact posture of the subject, etc. This was accounted for by linear scaling of the features for each recording separately by which the relative change of power is considered rather than the absolute.

As shown in Fig. 13, the variation between the subjects in the test set was quite high. The meal detection worked better for those with a clear audible change of bowel activity. Some of the less profound variations could be caused by posture adjustments which resulted in periods with more or less tight contact between microphone and skin. Such artifacts could also be the reason for two distinct periods with increased power that were observed in some recordings before and after the meal. One recording suffered from significant background noise that should be prevented by suitable shielding and grounding.

All selective decisions were made based on the performance of meal detection, except for the correlation-based feature selection method (c.f. Section IV-C, Step 1b). The latter method is a computationally cheap alternative to classical greedy/wrapper algorithms. To the degree that this method is suboptimal, it yields a conservative result with respect to overall system performance, and therefore does not jeopardize our qualitative conclusion with respect to the feasibility of sound based meal detection. Greedy feature selection methods such as forward selection and backward elimination should be considered in future work.

In future work, long-term recordings covering several meals and digestive states should be analyzed to validate general patterns that are correlated with the meal onset independent of the time of the day. Further states in the digestive cycle could potentially be defined as classes and contribute to detect meals with higher certainty. An extended feature space should be explored that could, for example, include the number and statistics of bowel sounds. Moreover, the type of ingested meals was not reported in this study. A first step would be to analyze whether one can differentiate between solid and liquid meals. Moreover, it should be investigated whether abdominal sound features can be identified that correlate with the ratio of carbohydrates, proteins and fats in the meal. The correlation of sound features with the increase of glucose concentration should be investigated. Such a correlation could be used to determine the insulin dose, either alone or in combination with meal size estimation based on continuous glucose monitoring [7].

A microphone would be a cheap and easily achievable add-on to an artificial pancreas. Relevant sounds for meal detection are chewing [30], swallowing [31] and bowel sounds [21]. Chewing and swallowing are obvious and early signs of ingestion. However, an (either subcutaneous or intraperitoneal) artificial pancreas will likely be located at the abdomen due to glucose sensing and insulin infusion. Monitoring bowel sounds has, therefore, the big advantage that the whole system can be more compact which will minimize the discomfort for the user. Thus, if future improvement of the presented method yields higher sensitivity and specificity, many patients will likely accept such a non-invasive feature that automatically detects meals in less than 15 min and improves the glucose regulation.Yes, but a better detection rate is required. The best suited abdominal position of the microphone with respect to high sensitivity towards an early and precise meal detection should be explored in future studies. Typical noise and artifacts occurring at the different locations and methods to filter contaminated sound should also be considered and further investigated. Furthermore, privacy concerns must be adequately addressed before a microphone can be included. Online sound processing can be performed on typical hand-held devices, while the classifier training may require higher computational power. The classifier model can be updated occasionally on a more powerful device using data that was collected before. To minimize the required storage capability, representative data sets can be stored in cloud-based solutions for this.

This pilot study showed that meals could potentially be detected based on bowel sounds. It is too early to conclude whether abdominal sound monitoring can be used as the only feature for meal detection, or rather as an add-on of meal detection based on CGM data. An increase of bowel sounds could, for example, confirm that a rising glucose concentration is caused by a meal. The design of a supervisory controller that fuses the information of sound and continuous glucose monitoring to detect meals and to determine insulin doses has not been part of this pilot study but is left for future work.

## VII. Conclusion

This feasibility study investigated the use of abdominal sound recordings for meal detection. An increased audible power occurs in the early postprandial period. This was exploited to train a classifier to recognize the digestion of a meal using features based on the power distribution in different frequency ranges. The experimental method of this study achieved a true positive rate with respect to meal detection of up to 0.5, which is not impressively high. However, false positive rates were consistently significantly lower, which indicates that abdominal sounds contribute with substantial, and early, information about meal intake.

Comparable methods based on continuous glucose monitoring in the subcutaneous tissue are reported to detect meals within 30 min to 40 min after the start of the meal, while this feasibility study in a limited population yielded a mean detection delay of 10 min. If an improvement like this can be realized with sufficiently high sensitivity and specificity, it would allow for prandial insulin being dosed (by an artificial pancreas) as much as half an hour earlier, which would significantly decrease postprandial glucose excursions and improve the overall glucose control. In the case of manual glucose control, the specificity of a meal reminder is less important.

These findings are strongly in favour of further investigation of sound based meal detection.
